# Optimizing patient derived mesenchymal stem cells as virus carriers for a Phase I clinical trial in ovarian cancer

**DOI:** 10.1186/1479-5876-11-20

**Published:** 2013-01-24

**Authors:** Emily K Mader, Greg Butler, Sean C Dowdy, Andrea Mariani, Keith L Knutson, Mark J Federspiel, Stephen J Russell, Evanthia Galanis, Allan B Dietz, Kah-Whye Peng

**Affiliations:** 1 Department of Molecular Medicine, Mayo Clinic, Rochester, MN 55905, USA; 2 Department of Laboratory Medicine and Pathology, Mayo Clinic, Rochester, MN 55905, USA; 3 Department of Obstetrics and Gynecology, Mayo Clinic, Rochester, MN 55905, USA; 4 Department of Immunology, Mayo Clinic, Rochester, MN 55905, USA; 5 Guggenheim 18, Mayo Clinic, 200 First Street SW, Rochester, MN 55905, USA

**Keywords:** Mesenchymal stem cell, Virotherapy, Ovarian cancer, Safety, Efficacy, Optimization

## Abstract

**Background:**

Mesenchymal stem cells (MSC) can serve as carriers to deliver oncolytic measles virus (MV) to ovarian tumors. In preparation for a clinical trial to use MSC as MV carriers, we obtained cells from ovarian cancer patients and evaluated feasibility and safety of this approach.

**Methods:**

MSC from adipose tissues of healthy donors (hMSC) and nine ovarian cancer patients (ovMSC) were characterized for susceptibility to virus infection and tumor homing abilities.

**Results:**

Adipose tissue (range 0.16-3.96 grams) from newly diagnosed and recurrent ovarian cancer patients yielded about 7.41×10^6 ^cells at passage 1 (range 4–9 days). Phenotype and doubling times of MSC were similar between ovarian patients and healthy controls. The time to harvest of 3.0×10^8 ^cells (clinical dose) could be achieved by day 14 (range, 9–17 days). Two of nine samples tested had an abnormal karyotype represented by trisomy 20. Despite receiving up to 1.6×10^9 ^MSC/kg, no tumors were seen in SCID beige mice and MSC did not promote the growth of SKOV3 human ovarian cancer cells in mice. The ovMSC migrated towards primary ovarian cancer samples in chemotaxis assays and to ovarian tumors in athymic mice. Using non-invasive SPECT-CT imaging, we saw rapid co-localization, within 5–8 minutes of intraperitoneal administration of MV infected MSC to the ovarian tumors. Importantly, MSC can be pre-infected with MV, stored in liquid nitrogen and thawed on the day of infusion into mice without loss of activity. MV infected MSC, but not virus alone, significantly prolonged the survival of measles immune ovarian cancer bearing animals.

**Conclusions:**

These studies confirmed the feasibility of using patient derived MSC as carriers for oncolytic MV therapy. We propose an approach where MSC from ovarian cancer patients will be expanded, frozen and validated to ensure compliance with the release criteria. On the treatment day, the cells will be thawed, washed, mixed with virus, briefly centrifuged and incubated for 2 hours with virus prior to infusion of the virus/MSC cocktail into patients.

## Background

Ovarian cancer kills more women in the United States than any other gynecologic malignancy. In 2012, it is estimated that there will be 22,230 new cases of ovarian cancer and 15,500 women will die from their disease [1]. Due to the lack of obvious symptoms, most women present with advanced stage cancer at the time of diagnosis. Frontline therapy is surgical debulking followed by platinium-taxane based chemotherapy, however, the majority of patients relapse within 5 years [2,3]. Residual disease after initial surgery is the strongest predictor of survival of ovarian cancer patients [4]. In a study of 194 patients, it was reported that minimizing residual disease through aggressive surgical resection was beneficial, especially in patients with carcinomatosis [4].

Mesenchymal stem cells (MSC) are multipotent cells capable of differentiating into cell types of various lineages, including osteocytes, chondrocytes and adipocytes [5]. They can be isolated and expanded from the bone marrow, adipose tissues, cord blood and other tissues. Numerous preclinical and clinical studies are evaluating the potential of unmodified or genetically modified MSC as a therapy for graft versus host disease, Crohn’s disease, multiple sclerosis, tissue repair and as carriers of therapeutic proteins or oncolytic viruses [6-9].

Replication competent tumor selective viruses from diverse families are being developed as a novel form of cancer therapy [10,11]. These viruses are engineered to express therapeutic transgenes (e.g. granulocyte macrophage colony stimulating growth factor GM-CSF) to enhance host immune response against the cancer, to synergize with drug or radiation therapy (e.g. cytosine deaminase, herpes simplex virus thymidine kinase (HSV-TK), sodium iodide symporter (NIS) or reporter genes to permit noninvasive nuclear imaging of sites of viral gene expression (HSV-TK, NIS) [12-15]. Numerous viruses are in advanced phase II or III testing in a variety of cancers, either as a single agent or in combination with drugs or radiation therapy [10,11]. We are developing the live attenuated Edmonston strain of oncolytic measles virus as a virotherapy agent for ovarian cancer, myeloma, glioma and mesothelioma [16-20]. Various phase I clinical trials are in progress evaluating the safety and maximal tolerated dose of recombinant MV expressing reporter genes to enable real time monitoring of the profiles of viral gene expression using a secreted carcinoembryonic antigen (MV-CEA) or NIS (MV-NIS) [21,22]. A phase I dose escalation trial in which MV-CEA (10^3^ to 10^9^ TCID_50 _virus) was given to patients with Taxol and platinum-refractory recurrent ovarian cancer was recently completed [23]. Twenty-one patients were treated with MV-CEA delivered intraperitoneally via a catheter every 4 weeks for up to 6 cycles. The virus was well tolerated with no dose-limiting toxicity or virus induced immunosuppression. Five patients had significant decreases in CA-125 levels, and median survival of patients on study was 12.15 months (range, 1.3-38.4 months), which compares favorably to an expected median survival of 6 months in this patient population [24]. There was dose-dependent CEA elevation in peritoneal fluid and serum, suggesting that there was viral replication, albeit low, in the patients. We have hence extended our Phase I trial to include the evaluation of MV-NIS as a single agent (10^8 ^and 10^9^ TCID_50 _per dose, 6 cycles) in ovarian cancer patients with recurrent disease [25]. The NIS gene encoded by the MV-NIS virus enables the infected cell to concentrate radioisotopes (e.g. Tc-99 m pertechnetate, I-125), thus enabling SPECT-CT imaging of the sites and dosimetric measurements on the level of viral gene expression. The MV-NIS Phase I trial (Clinicaltrials.gov number NCT00408590) was recently completed and closed for data analysis (Galanis, unpublished data).

To further improve viral delivery, we and others have been exploring the use of cells as carriers of oncolytic viruses to tumors [9,26-31]. Indeed, virus loaded cell carriers have potential advantages as a hybrid form of cellular and oncolytic virotherapy. Mesenchymal stromal cells home to sites of inflammation or wound healing and are being investigated preclinically and clinically for tissue regeneration or as vehicles for therapeutic proteins and viruses [32]. In particular, MSC appear to be highly effective as virus carriers to cancer. Using passively immunized mice given an intraperitoneal dose of antiviral antibodies, we have demonstrated that adipose tissue derived MSC from healthy donors can be infected by MV, home to ovarian tumor xenografts in athymic mice and exert a superior therapeutic outcome compared to virus alone [33]. In preparation for clinical testing of this approach, we evaluated here the feasibility of expanding MSC from ovarian cancer patients, 6 recurrent and 3 newly diagnosed, and compared their migration and tumor homing abilities with MSC from healthy donors. In particular, we were interested to evaluate if NIS imaging could enable us to monitor trafficking of the MSC *in vivo* and the speed at which the cells are able to co-localize with the ovarian tumors. Finally, we also optimized the MSC-virus loading protocols and evaluated the safety and efficacy of using MV infected MSC in passively immunized animals.

## Results

### Feasibility of harvest and expansion of MSC from ovarian cancer patients

The ovMSC were generated from adipose tissues surgically obtained from 6 newly diagnosed and 3 recurrent ovarian cancer patients scheduled for ovarian cancer resection by laparotomy. Following the usual midline skin incision, approximately 2 cm^3 ^of subcutaneous fat was excised prior to incision of the fascia. Following confirmation of hemostasis, the prescheduled procedure was continued. The adipose tissues were placed into sterile tubes and processed within 2–5 hours. Healthy donor MSC were obtained from surgical wastes and frozen in liquid nitrogen as previously described [34].

Mesenchymal stem cells were successfully isolated from adipose tissues obtained from all nine ovarian cancer patients (Table 1). The amount of fat collected varied significantly from less than 0.2 gm up to almost 4 gm. It is very difficult to count cells from newly processed fat. However, after culture establishment all samples had similar growth kinetics with population doublings approaching one per day similar to our previous report [34]. We saw no significant differences in the growth kinetics using fat from newly diagnosed or relapsed patients. We also note that cultures were successful even using the smallest amount of fat collected. In four samples (including one sample that was split), we delayed processing to simulate real world clinical experiences, where samples often require additional steps (i.e. travel time or pathology). While not powered for statistical analysis, we noted that the growth kinetics were also similar between these and those processed immediately.

**Table 1:** Information on mesenchymal stem cells derived from ovarian cancer patients

**Sample ID**	**Disease**	**Process timing**	**Grams of Fat Processed**	**Karyotype (see footnote)**
1	Newly Diagnosed	Immediate	0.95	Normal
3	Newly Diagnosed	Immediate	0.16	Abnormal^1^
4	Newly Diagnosed	Immediate	2.26	Normal
7	Newly Diagnosed	Immediate	3.96	Normal
8	Newly Diagnosed	43 hours	3.40	Normal
10	Newly Diagnosed	Immediate	0.80	Normal
2	Relapsed	14 hours	2.23	Normal
5a	Relapsed	Immediate	1.50	Normal
5b	Relapsed	20 hours	1.60	Abnormal^2^
6	Relapsed	11 hours	1.79	Normal

^1^Of 20 metaphases, 16 were normal and 4 had trisomy 20.

^2^Of 30 metaphases, 28 were normal and 2 had trisomy 20 and a balanced three-way translocation involving chromosomes 1, 10 and 13.

Adipose tissues were either processed immediately or stored in the refrigerator at 4°C before collagenase digestion. Expanded MSC were karyotyped and two out of ten samples were found to have abnormal karyotype.

**Figure 1:**
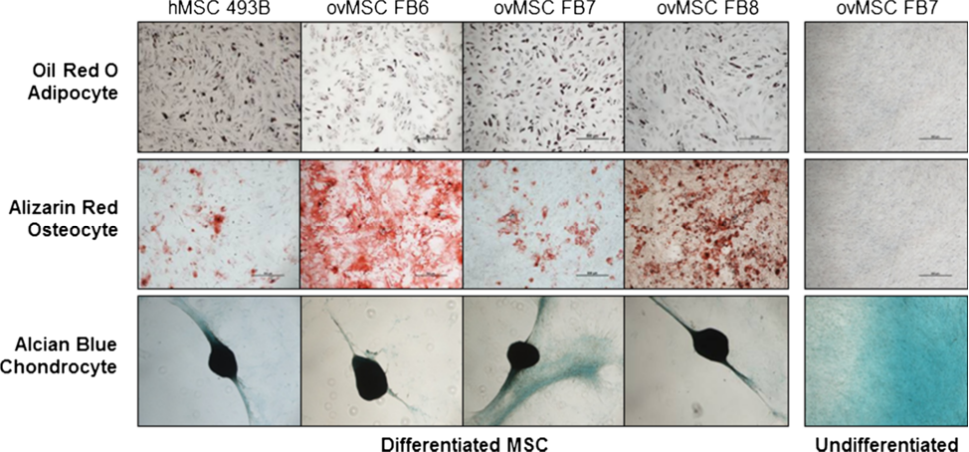
**Ovarian cancer patient derived MSC (ovMSC) retains multi-potent lineage.** Representative results of in vitro differentiation of MSC into adipocytes, osteocytes and chondrocytes post culture in differentiation media. Respective stains used are as indicated. hMSC: healthy donor MSC.

The ovMSC have a phenotype that is characteristic of MSC, that is, they were positive for CD73 (99.9% of population), CD90 (99.9%), CD105 (98.5%), CD44 (100%) and HLA-ABC (99.0%). They are negative for CD14 (0.8%), CD45 (1.4%) and HLA-DR (0.8%). The ovMSC population doublings per day were 1.3 (passage 2), 1.2 (passage 3), 1.2 (passage 4) and 0.9 (passage 5), comparing favorably with those of hMSC [34]. The differentiation potential of ovMSC into adipocytes, chondrocytes and osteocytes was also confirmed, respectively, by Oil-Red-O, Alcian blue and Alizarin Red staining (Figure 1). Finally, samples were analyzed using karyotype analysis to look for chromosomal alterations. Two of our samples showed karyotypic abnormality. Surprisingly, the abnormality was similar in both cases (trisomy 20).

### Optimizing protocols for MV infection of MSC

**Figure 2:**
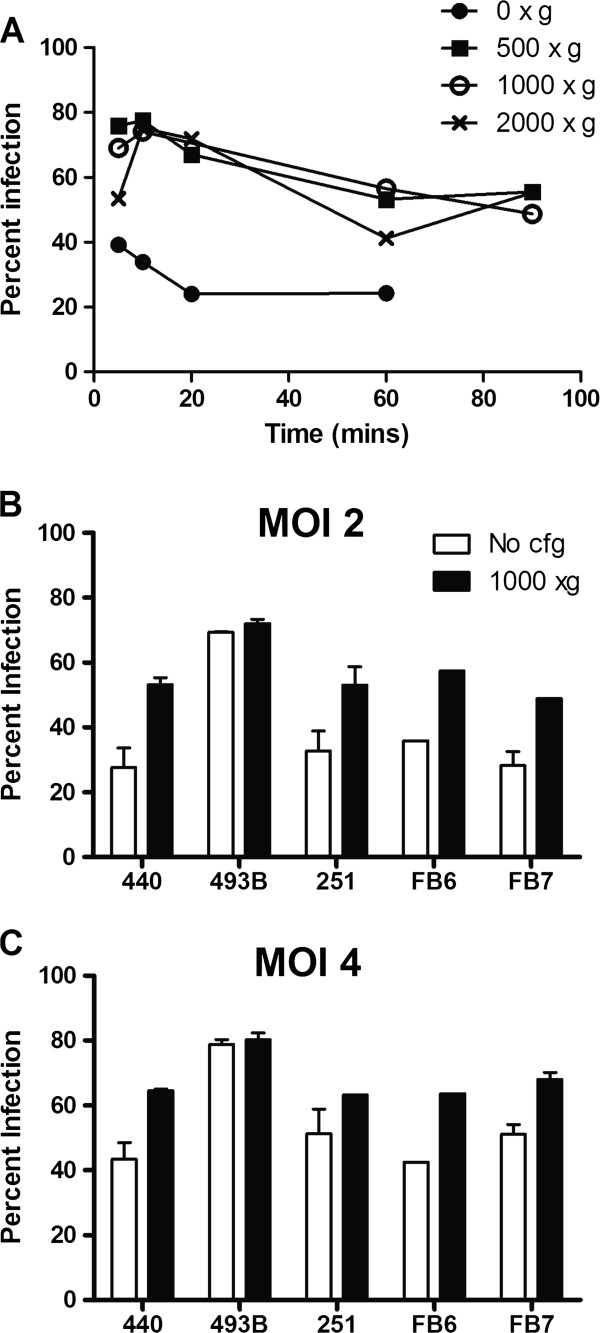
**Centrifugation improves MV infectivity of MSC.** (**A**) MV-GFP and hMSC were centrifuged at the speeds and times indicated prior to a 2 h incubation period. Percentage of GFP positive cells were analyzed 48 h later. (**B**, **C**) Percentage of MV-GFP infected MSC from healthy donors (440, 493B, 251) or ovarian cancer patients (FB6, FB7) at MOI of 2 (**B**) or 4 (**C**). 1000 xg centrifugation for 10 mins. Representative data from duplicate experiments.

Various physical methods were explored to increase virus infection or gene expression in hMSC. Cells either underwent heat shock treatment at 42°C before virus loading, were exposed to DMSO (incubation with 4% DMSO in standard media before, during or after MV infection) or centrifuged with the virus inoculum (500, 1000 or 2000 xg) prior to the standard 2 h virus-cell incubation period at 37°C. Compared to untreated cells, heat shock and DMSO treatments did not significantly improve numbers of MV-infected GFP positive cells (data not shown). In contrast, centrifugation of virus with MSC prior to the 2 h incubation period enhanced MV infectivity (Figure 2A). Infectivity was increased from 40% (no centrifugation) to 70% with 500×g or 1000×g centrifugation. A brief centrifugation of 5–10 minutes was optimal with no compromise on cell viability. Longer centrifugation times negatively impacted this enhancement in infectivity, presumably because cell viability was compromised (Figure 2A).

Frozen ovMSC stocks from 3 ovarian cancer patients (FB6, FB7, FB8) and 3 healthy donors (440, 439B, 251) were randomly selected for further analysis. The cells were infected with MV-GFP at MOI of 2 or 4, with and without centrifugation (1000xg, 10 min) prior to the incubation period. The percentage of GFP positive MSC was determined by flow cytometry 48 h later (Figure 2B). As expected, the numbers of infected cells increased with increasing amounts of virus with peak infectivity at MOI 4.0. There was variability in donor cell susceptibility to MV infection. Regardless of the source of MSC (healthy donor versus ovarian cancer patients), some MSC had intrinsically poorer infectivity (30%) compared to the others (70%).

### Comparing migration of ovMSC and hMSC to ovarian cancer cells

**Figure 3:**
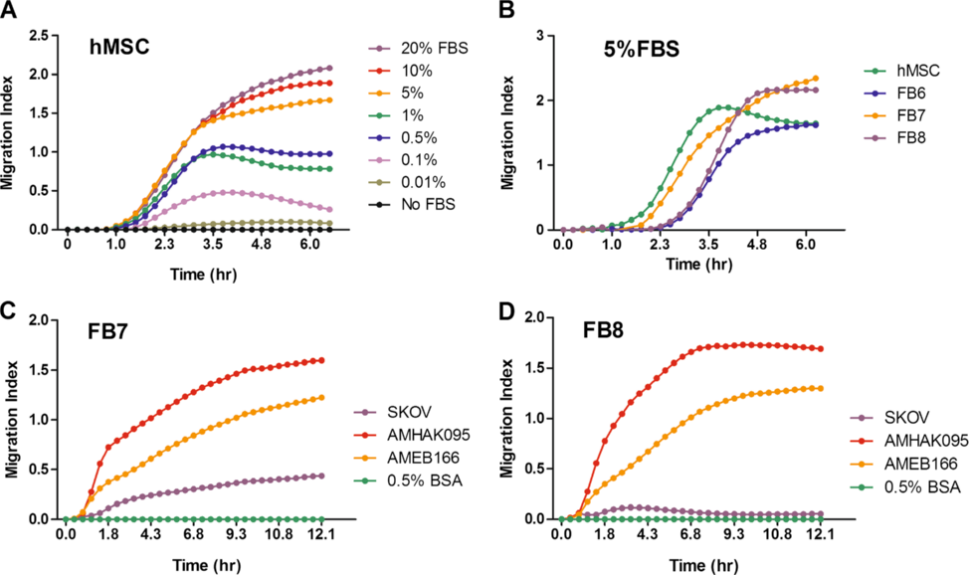
**Dynamic real time monitoring of MSC migration using the xCELLigence chemotaxis assay.** (**A**) The extent of hMSC migration is dose dependent on the concentration of fetal bovine serum (FBS) in the media. FBS concentration ranged from no FBS to 20% FBS in alpha-MEM. (**B**) Migration of hMSC or ovarian MSC (FB6-8) towards alpha-MEM supplemented with 5% FBS. Migration of ovMSC (**C**) FB7 or (**D**) FB8 towards conditioned media harvested from SKOV3ip.1 ovarian cancer cell line or from primary ovarian cancer cells isolated from clinical specimens (AMHAK095, AMEB166). Representative data from duplicate experiments.

The Roche xCELLigence system was used to monitor the dynamic migration of MSC towards chemoattractants in real time over 12–48 hours. The system was first validated using media supplemented with fetal bovine serum (FBS). There was minimal migration of MSC towards media supplemented with no FBS (Figure 3A, B) or 0.5% BSA (Figure 3C). As expected, the rate and extent of MSC migration is dependent on the concentration of the chemoattractant added. With increasing amounts of FBS, there was a corresponding increase in the rate and numbers of MSC that migrated across the transwells within 4 hours of exposure (Figure 3A).

To compare the migration abilities of hMSC and ovMSC *in vitro*, 5% FBS in media was used as a chemoattractant. As shown in Figure 3B, ovMSC and hMSC migrated at comparable rates and no detectable migration occurred in the absence of FBS. Migration of MSC towards conditioned media from a human ovarian cancer cell line, SKOV3ip.1, and conditioned media from primary ovarian cancer cells from surgical samples (AMHAK095, AMNMC313, AMEB166), was also tested. The rates of MSC migration towards SKOV3ip.1 or primary ovarian cancer cells were plotted and found to be comparable for hMSC and ovMSc (Figure 3C). For most MSC, migration reached steady state within 3 hours of exposure to the conditioned media.

### Homing of hMSC and ovMSC to ovarian tumor xenografts

**Figure 4:**
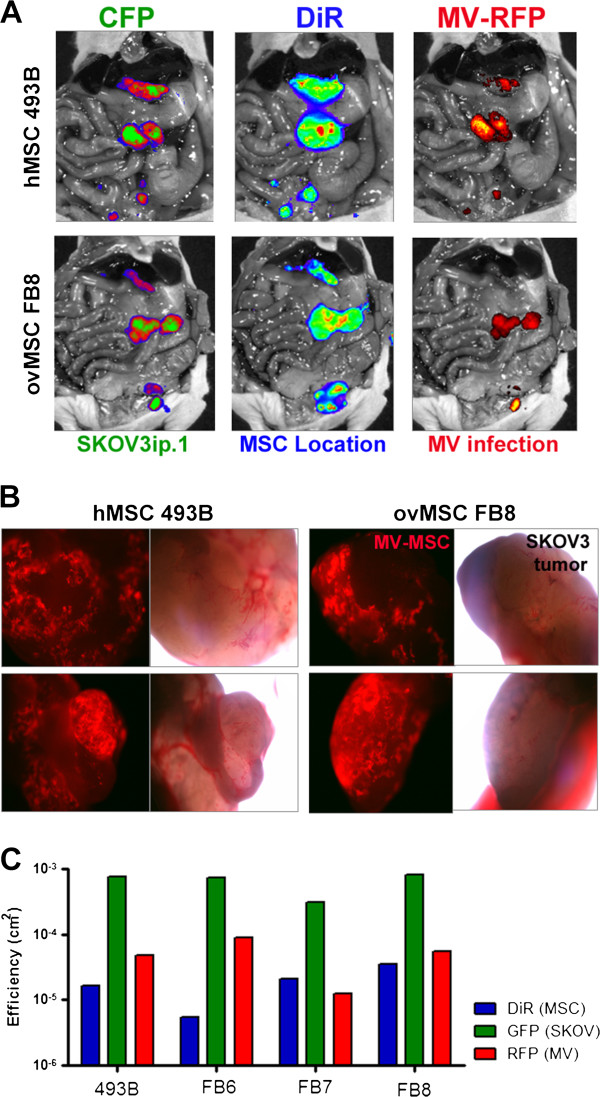
**Multimodality imaging showing co-localization of MV infected MSC with peritoneal human ovarian xenografts post intraperitoneal administration.** (**A**) SKOV3ip.1 tumors were stably expressing cyan fluorescent protein (CFP), MSC were labeled with DiR, and infected with measles virus expressing red fluorescent protein (MV-RFP). Representative images from mice that received MSC from healthy donor (hMSC 493B) or ovarian cancer patient (FB8) showed good co-localization of MV infected MSC with the tumors. (**B**) Tumors from mice were harvested at day 10 and examined for RFP expression. MV-RFP infected MSC were found on the tumors. Images of tumor nodules were taken at 40X magnification under a fluorescence microscope with a green filter or under bright-field. (**C**) Quantitative measurement of the amount of CFP (tumors), DiR (MSC) or RFP (MV infection) fluorescence in mice given MV infected hMSC or ovMSC.

We previously demonstrated that hMSC home to ovarian tumors post i.p. administration into mice with SKOV3ip.1 xenografts [33]. To confirm that ovMSC can co-localize with ovarian tumors and deliver oncolytic MV, athymic mice with omental and disseminated peritoneal SKOV3ip.1 tumors were given MSC440 or ovMSC (FB6, FB7 or FB8) i.p. at 7 days post-implantation. Mice were harvested on days 1, 3, and 10 post MSC administration. Multimodality imaging was used to identify the various components; cyan fluorescent protein (CFP) was used to identify the SKOV3ip.1 cells, lipophilic DiR dye was used to label MSC for fluorescence imaging at 756 nm, and oncolytic MV-RFP expresses RFP to facilitate identification of areas of viral gene expression. As shown in Figure 4, there was good co-localization of DiR labeled MSC with the CFP positive tumor nodules and RFP expression from MV infection. Measles virus infected ovMSC clearly were able to home to SKOV3ip.1 tumor nodules.

### MSC tumorigenicity study in SCID beige and athymic mice

**Figure 5:**
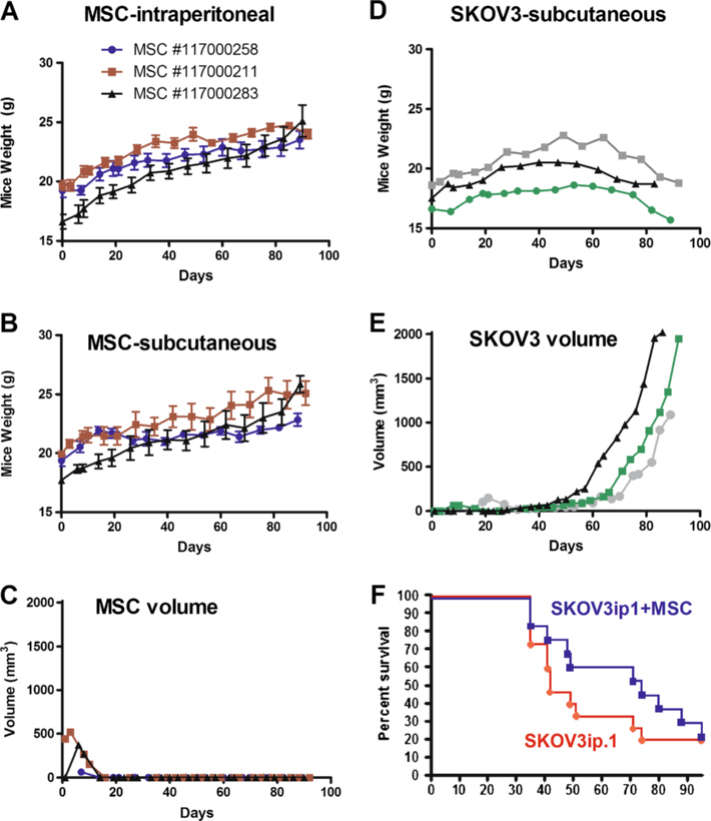
**Safety study to evaluate the tumorigenic potential of MSC in beige SCID and athymic mice.** MSC from three different healthy donors (MSC lot numbers as indicated) were cultured deliberately to more than 25 passages. Mice received 4×10^7 ^MSC/500 μl saline intraperitoneally (n = 6 mice per cohort MSC) or subcutaneously (2×10^7 ^MSC/100 μl saline, n = 3 mice/cohort of MSC). Body weights of mice that received (**A**) intraperitoneal or (**B**) subcutaneous MSC were recorded at regular intervals. (**C**) In the subcutaneous model, size of the ‘lump’ formed from the MSC deposit was recorded in two dimensions and volume calculated. (**D**) Body weights of the control mice that received SKOV3 tumor cells (2×10^6 ^cells/100 μl). (**E**) Growth of SKOV3 subcutaneous tumor in control mice. (**F**) Survival curve of athymic mice given 10^6 ^SKOV3ip.1 (n = 15 mice) or a cocktail of 10^6 ^SKOV3ip.1 and 10^6 ^MSC (n = 13 mice) intraperitoneally.

To determine if MSC are tumorigenic, SCID beige mice were injected either subcutaneously or i.p. with the maximum feasible number of hMSC which had been deliberately passaged for extended periods in culture. Three separate lots of hMSC were collected and purified from adipose tissues of three different healthy donors. These hMSC had been cultured for more than 25 population doublings, representing potential cell expansion to more than 10^12 ^cells. Mice were injected subcutaneously with 2 × 10^7 ^MSC or 4 × 10^7^ i.p. As a positive control, some mice received SKOV3 human ovarian tumor cells. Mice were monitored for signs of tumor formation due to hMSC administration. All mice (n = 3 per MSC cohort) that received hMSC subcutaneously showed no signs of tumor development at the end of the study (90 days). Their body condition remained excellent, no adverse clinical signs were seen and all mice continued to gain weight during the study (Figure 5). In contrast, control mice receiving SKOV3 developed tumors at the injection site. Their body condition was fair, but the effect of the tumor growth was evident from their body weight measurements showing minimal to no gain in body weight (Figure 5). Mice that received hMSC i.p. showed no signs of ill health (n = 6 per MSC cohort). Their body weight continued to increase, mice appeared healthy and remained active throughout the study. At the end of the study, mice were examined for the presence of tumors or growths. While no tumors were seen, a small white colored deposit was seen either on the omentum or pancreas in all mice given hMSC i.p. In some instances, these deposits were found as an adhesion to the peritoneal wall or inguinal fat pad. Histology was performed on the deposits and through H&E and Masson-Trichrome staining, these deposits appear to be acellular connective tissue which stained positive for collagen with Masson-Trichrome (data not shown).

To determine if MSC promote tumor growth, athymic mice received MSC, SKOV3ip.1 (n = 15 mice) or a cocktail (1:1 ratio) of MSC and SKOV3ip.1 cells (n = 13 mice). MSC given at the same time as tumor cell implantation did not promote tumor growth (Figure 5). There was no apparent difference in clinical symptoms (e.g. ascites formation, jaundice, weight loss) noted in mice from the treatment groups. Survival curves of both treatment groups were not significantly different from each other (p = 0.3670, log-rank Mantel-Cox test).

### NIS mediated SPECT-CT imaging of MSC co-localization with tumors *in vivo*

**Figure 6:**
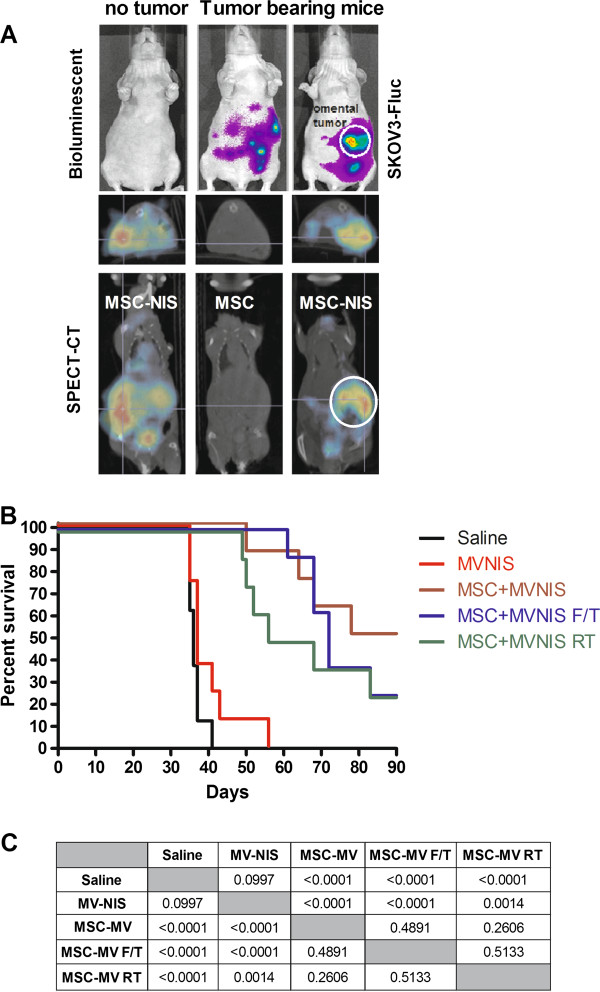
**Antitumor activity of MV-NIS or MV-NIS infected MSC in athymic mice passively immunized with human measles immune sera.** (**A**) Bioluminescent and SPECT-CT imaging show rapid co-localization of Tc-99 m-pertechnetate loaded MV-NIS infected MSC (MSC-NIS) with luciferase positive SKOV3ip.1 tumors. (**B**) Mice with SKOV3ip.1 tumors were passively immunized with measles immune human sera and given 10^5 ^TCID_50 _MV-NIS or 10^5 ^MV-NIS infected hMSC at 7 days post-tumor implantation. RT = MSC were given 20 Gy radiation immediately before MV-NIS infection. F/T = Frozen stock of MV infected MSC was thawed, washed and used immediately. Actively growing = MSC were in log phase of expansion prior to MV-NIS infection. (**C**) p values comparing the survival curves of the respective groups are indicated (Log rank test, Mantel Cox).

NIS expression enables the cell to concentrate radioisotopes (e.g. Tc-99 m pertechnetate, radioiodine) against the concentration gradient, thereby enabling noninvasive imaging of sites of MV-NIS gene expression [25,35]. Here, we exploited this technology to monitor the kinetics of MSC trafficking after i.p. administration into tumor bearing mice. Location of the implanted ovarian tumors was detected using bioluminescent imaging for SKOV3ip.1-FLuc tumor nodules (Figure 6). The MSC were infected with MV-NIS for 24 hours after which the cells were loaded with I-125 and given to mice. The amount of radioiodine uptake was about 25 μCi per 10^6 ^MV-NIS infected MSC.

No radioactive signals were seen in the tumor bearing mouse that received uninfected MSC as the cells did not concentrate I-125 (0.2 μCi per 10^6 ^MSC). In contrast, strong radioactive signals were seen in mice that received MV-NIS infected MSC (24–26 μCi per 10^6 ^MV-NIS/MSC). In non-tumor bearing mice, the radioactive signals were more diffused around the peritoneal cavity (Figure 6). Bioluminescent imaging revealed the presence of omental tumors and some disseminated peritoneal tumors in tumor bearing animals (Figure 6). Through SPECT-CT imaging, we detected rapid localization of the I-125 pre-loaded MV-NIS/MSC cells at the omental tumor and peritoneal tumors, showing good concordance with the bioluminescent tumor images (Figure 6). Since these SPECT-CT images were taken within 5–8 minutes of cell infusion, it is apparent that the rapid localization of MSC with tumors is perhaps more dependent on cell-to-cell contact interactions rather than chemotactic attraction in this setting.

### Optimizing *in vivo* delivery of MV by MSC

The optimal MSC platform for MV delivery was evaluated in athymic mice bearing orthotopic intraperitoneal SKOV3ip.1 tumors. Since most ovarian cancer patients enrolled in the clinical trial would be measles immune either from previous vaccination or natural measles infection, mice were passively immunized by i.p. administration of pooled measles-immune human serum (50 EU per mouse) to mimic the measles immune status of the patients. Mice were then given equal numbers (10^5^) of MV or MV infected MSC. Three different MSC platforms were tested; using actively growing MSC infected with MV-NIS, using freshly thawed MSC that had previously been infected with MV-NIS for 2 h prior to freezing, or using MSC that had been lethally irradiated (20 Gy) prior to infection with MV-NIS. For all MSC groups, virus infection was performed at MOI 4.0, with a brief centrifugation prior to the 2 h incubation period to improve virus infection (Figure 2A), after which the cells were washed and either used immediately (actively growing or irradiated groups) or frozen for storage in liquid nitrogen (freeze/thaw group). To ensure neutralization of surface bound virus, the MSC were incubated *in vitro* with measles-immune serum (50 EU) for 30 minutes at 37°C before injection in the animals. Thus, each mouse received a total of 100 EU of measles immune human sera.

The Kaplan Meier survival curves of mice and the p values comparing differences between treatment groups are shown in Figure 6. The median survival for saline control was 36 days (n = 8 mice), MV was 37 days (n = 8 mice), MV/MSC (actively growing) was 84 days (n = 9 mice), MV/MSC (freeze/thaw) was 72 days (n = 8), MV/MSC (RT) was 62 days (n = 8). ‘Naked’ MV in measles immune animals has no therapeutic activity (Figure 6). All but one mouse in the saline and MV groups (15 out of 16) were euthanized because they developed bloody ascites, with extensive dissemination of ovarian tumors in the peritoneal cavity, perigastric area, and on the peritoneal side of the diaphragm. In contrast, survival curves of mice treated with all three MV/MSC platforms were significantly extended compared to MV or saline. In contrast to the saline and MV groups, only 28% of mice in the MV/MSC, MV/MSC freeze/thaw, and MV/MSC RT groups developed ascites (7 of 25). Examination at necropsy indicated tumor obstruction or constriction of the gastrointestional tract around the gall bladder or bile duct as the cause of euthanasia of these mice. The remaining animals were euthanized due to weight loss of more than 20%. At the conclusion of the study at day 90, a number of mice from all the MSC/MV treatment groups were still alive and appeared healthy (9 of 25).

## Discussion

Oncolytic measles virus has promising antitumor activity and is being investigated as an experimental cancer therapeutic in various Phase I clinical trials [21,36]. In athymic mice bearing intraperitoneal ovarian cancer, we showed that repeat dosing of a recombinant oncolytic measles virus expressing the soluble carcinoembryonic antigen (MV-CEA) virus resulted in significant extension in survival of mice [19,37]. A Phase I clinical trial in which MV-CEA was given i.p. to patients with recurrent ovarian cancer was recently completed [23]. Dose limiting toxicity was not reached in this dose escalation trial in which 6 cycles of 10^3^ to 10^9 ^TCID_50 _of MV-CEA was given [23]. The best objective response was dose-dependent disease stabilization in 14 of 21 patients with a median duration of 92.5 days (range, 54–277 days). Median survival of patients on study was 12.15 months (range, 1.3-38.4 months).

To improve therapeutic outcome using MV therapy, we focused on maximizing delivery of the virus by using tumor homing cell carriers. We evaluated numerous cell types, including IL-2 expanded T cells [38], CD14+ derived monocytes [39], but chose adipose tissue derived MSC due to their ability to be infected by MV and co-localization with ovarian tumors. In contrast to naked virus, these MV infected MSC significantly extended the survival of measles immune tumor bearing mice [33]. There are also well established standard operating procedures in place for harvest, isolation, culture, expansion and banking of adipose tissue derived MSC in the Human Cell Therapy Laboratory of our institute (A. Dietz). The feasibility and safety of using autologous or allogeneic MSC in humans has been confirmed in a number of phase II and III studies. For example, infusion of autologous MSC in patients with ischemic stroke (2 doses, 5×10^7 ^cells/dose, intravenous) or multiple sclerosis and amyotrophic lateral sclerosis (6×10^7 ^cells intrathecally or intravenously) or allogeneic MSC to patients with myocardial infarction (0.5 to 5 ×10^6^/kg intravenous) showed that autologous or allogeneic MSC can be safely administered and tolerated in humans [40-42].

A potential concern with MSC administration is that the cells could be tumorigenic or promote the growth of tumors. Since MSC used in our study will be infected by a replicating virus, the cells typically die within 5 days from virus infection [33]. In safety studies using athymic mice given a cocktail of ovarian cancer cells and MSC, we showed here that the survival of mice was not significantly different from mice that received tumor cells only. Immunocompromised beige SCID mice were also implanted with large numbers of MSC (4×10^7 ^cells/25 gram mouse, 1.6×10^9^/kg) that had been deliberately passaged in culture for more than 25 passages, representing cell expansion to more than 10^12 ^cells. No tumor growth was seen in mice given MSC subcutaneously or i.p. when necropsied at the end of 90 days, concurring with results from other published reports [43]. In mice that received large numbers of MSC i.p., a white acellular tissue was seen in the omentum or pancreas, sometimes as an adhesion to the peritoneal wall or inguinal fat pad. Histological analysis using H&E staining and Masson-Trichrome staining revealed that these deposits were acellular connective tissue composed of collagen (positive staining for collagen with Masson-Trichrome stain). The significance of the ‘connective tissue’ is not known, but since MSC home to the omentum post i.p. delivery, we suspect that the deposit represents remnants of the extracellular matrix from the large numbers of cells that have homed to the omentum and died eventually.

Results of this study confirmed that it is highly feasible to isolate and expand MSC from ovarian cancer patients, can be infected by MV, home and deliver the virus to tumor nodules in athymic mice. For our clinical trial, we plan to use autologous patient derived MSC to deliver oncolytic MV in this first-in-human study. The MSCs have a population doubling time that is comparable to MSC from healthy donors and we aim to expand and bank sufficient cells for repeat cycles of treatment (aiming to bank >10^9 ^cells per patient). One puzzling finding in our study was the abnormal karyotype of trisomy 20 in the expanded MSC from two of the nine patients. In total, we have tested the karyotype in more than 20 individuals with a variety of diseases including amyotropic lateral sclerosis, Type I diabetes, critical limb ischemia without detection of karyotypic abnormality. Thus, this finding of trisomy 20 in MSC of two ovarian cancer patients is puzzling. Since we did not test the MSC karyotype at harvest, it is not clear if the cells acquired trisomy 20 during expansion in culture. The three instances of viable trisomy are trisomy 21 (Down’s syndrome), trisomy 13 (Patau syndrome) and trisomy 18 (Edward’s syndrome). Complete trisomy 20 is not viable but trisomy 20 mosaicism is estimated to occur in 1 out of 7,000 pregnancies detected prenatally from amniocentesis or chorionic villus sampling [44]. However, the outcome is normal in 90-93% of prenatally diagnosed cases. Trisomy 20 were noted in non-cancer smokers and mine workers where it was shown they universally harbor increased numbers of abnormal cells within their airway epithelium [45,46]. Approximately 50% of ovarian tumors and cell lines have copy number increases in 20q [47], genes amplified therein, including EEF1A2, may play a central role in the pathogenesis of sporadic and hereditary ovarian carcinoma [46]. Currently, the significance of trisomy 20 in the expanded MSC of the ovarian cancer patients is not clear but will be the subject of further investigations. In the event that the expanded MSC from the clinical trial fail to meet the release criteria of normal karyotype, the patient will not receive MSC/MV therapy but virus alone.

In this study, we also explored the feasibility of using virus-preinfected cells as the frozen cell bank. In vitro assays indicated that the virus infected cells will proceed to express the viral genes and progeny post thaw from cryopreservation in liquid nitrogen. As shown in this report, actively growing MV-infected MSC and MV infected MSC frozen/thawed cells have comparable antitumor activities. However, since 10^9 ^TCID_50 _MV was well tolerated in our Phase I trial with preliminary signs of activity, we are proposing to deliver the cocktail of infected cells with the 10^9 ^TCID_50 _of virus inoculum. Hence, our planned delivery strategy will involve thawing of certified MSC, mixing with our GMP grade virus on the day of treatment, a 5 minute low speed centrifugation, incubation for 2 hours at 37°C, followed by infusion of the cell-virus mixture into the patient by a catheter in the peritoneal cavity. In addition to MSC, other cells types are being developed as carriers of proteins or viruses [9,26]. Neural stem cells are being evaluated as carriers of oncolytic adenovirus for glioma therapy [48,49] and cytokine induced killer cells for delivery of oncolytic vaccinia virus [50]. It is hoped that by using cell carriers that home to tumors in conjunction with the replication virotherapy, we could significantly amplify the initial payload of cytotoxic agent to achieve significant improvements in the outcome of cancer therapy.

## Materials and methods

### Isolation and culture of MSC from adipose tissues

All procedures involving human subjects were reviewed and approved by the Mayo Clinic Institutional Review Board (IRB). Human adipose tissues were obtained from healthy donors as fat biopsies in an outpatient clinic. Adipose tissues were also harvested from patients undergoing surgery for ovarian cancer resection via laparotomy. Following the usual midline skin incision, 2 cubic cm of subcutaneous fat was excised prior to incision of the fascia. The fat tissues were minced with surgical scalpels and incubated in 0.075% collagenase type I (Worthington Biochemical, Lakewood, NJ) for 90 min at 37°C. Digested tissue was centrifuged at 400 g for 5 min with the pellet washed in PBS, passed through a 70 μm cell strainer (BD Biosciences, San Jose, CA), and incubated in red blood cell lysis buffer (154 mM NH4Cl, 10 mM KHCO3, 0.1 mM EDTA). Cells were grown in T-175 cm^2 ^flasks at a concentration of 1.0-2.5 ×10^3 ^cells/cm^2 ^in Advanced MEM with 5% PLTmax (Mill Creek Life Sciences, Rochester, MN), 100 U/ml penicillin, 100 g/ml streptomycin, and 2 mM L-glutamine (Invitrogen, Carlsbad, CA, USA) in a 37°C 5% CO_2 _incubator for 3–4 days. When cells were 60-80% confluent, they were passaged using TrypLE (Trypsin Like Enzyme, Invitrogen). Cells were frozen in aliquots in liquid nitrogen and stored until use. Only low-passage cells (P5-7) were used for all the experiments of this study, with the exception of the tumorigenicity study. These cells were phenotyped for typical MSC cell markers, and were negative for Class II, CD14, CD45, HLA-DR, and positive for CD44, CD73, CD90, CD105, and Class I (A. Dietz, unpublished data).

### Viruses, lentivectors and cell lines

Recombinant Edmonston strain measles virus expressing Firefly Luciferase (FLuc), red fluorescent protein (RFP), green fluorescent protein (GFP), or human sodium iodide symporter gene (NIS) were generated as described previously [20]. Briefly, African green monkey Vero producer cells were infected with the viruses at MOI 0.02 and when 90% of the cells were in syncytia, the supernatant was removed and the cells were scraped into reduced serum medium, Opti-MEM (Invitrogen). The cell pellet was frozen and thawed twice and clarified by low speed centrifugation for 5 minutes. The viral supernatants were frozen at ≤ 65°C until use. Measles viral titers were determined by TCID_50 _titration on Vero cells. To generate the lentivectors, 293 T cells were co-transfected with gag-pol expression plasmid pCMV8.91, VSV.G envelope expression plasmid pMD-G, and vector plasmid encoding FLuc, RFP, GFP or NIS [51]. Vector supernatant was collected 48 hours later, filtered (0.45 um) and frozen at ≤ 65°C until use. Human ovarian cancer cells, SKOV3ip.1, stably expressing FLuc were maintained in Alpha-MEM (Lonza, Allendale, NJ) supplemented with 20% fetal bovine serum (FBS, Gibco), 100 U mL-1 penicillin-streptomycin, and 2 mM L-glutamine. Adipose derived mesenchymal stem cells were maintained in Advanced MEM (Invitrogen, Carlsbad, CA) supplemented with 5% PLTmax, 2 U mL-1 heparin, 100 U mL-1 penicillin-streptomycin, and 2 mM L-glutamine.

### MSC x-CELLigence migration assay

Cells were grown in serum-free medium for 24 hours (OptiMEM with 0.5% BSA). Pre-warmed media, with or without chemoattractant, was added to the lower chambers of the CIM-Plate 16 (Roche Applied Bioscience, Indianapolis, IN) according to manufacturer’s instructions. Serum-free medium was placed in the top chamber to hydrate the membrane. The plate was placed for 1 hour in the incubator to obtain a background measurement. MSC (2 × 10^4 ^cells) were added in the upper chamber and the cells were allowed to settle to the bottom. Measurements were taken every 5 minutes. To collect conditioned media, normal culture media was removed from cancer cell lines and replaced with OptiMEM with 0.5% BSA for 48 hours after which the conditioned media was collected and stored at −80°C until time of use.

### Virus Infection and flow cytometry

MSC were infected in serum-free media (Opti-MEM) with MV-GFP for 2 hours at 37°C while in constant rotation to prevent cells from settling. Cells were infected at multiplicity of infection (MOI) of 2 or 4. After incubation, MSC were gently pelleted and supernatant removed. Cells were plated and allowed to incubate at 37°C for 48 hours. Percent infect was determined by flow cytometry on a FACScan (BD Dickinson, Franklin Lakes, NJ) with a blue laser (excitation 488 nm). To prepare cells for analysis, they were collected by centrifugation and washed in PBS. The cells were then resuspended in PBS containing 4% paraformaldehyde (Sigma, St. Louis, MO). The cells were then incubated for 30 min at room temp. Cells were washed twice in cold PBS, resuspended in PBS and incubated overnight at 4°C, until acquisition on the flow cytometer. On each sample, a minimum of 10,000 events was collected. Analysis of the data was performed with CELLQuest software (BD). To enhance virus infection, various physical methods were tested. For heat shock, MSC were incubated briefly in a 42°C water bath for 0, 5, or 10 minutes. Cells were cooled on ice for 5 minutes and virus was added and incubated as above. For DMSO treatment, MSC were treated with 4% DMSO 48 hours before, during, or after infection. To test the impact of centrifugation, virus was added and the mixture was centrifuged at 500, 1000, and 2000 × g for various times and upon completion of centrifugation, cell pellets were gently resuspended and the tubes were placed at 37°C on a rotator. After 2 hours, cells were plated in 12-well plates. MSC were allowed to incubate at 37°C for 48 hours and percent infect was determined by flow cytometry.

### In vivo experiments

All procedures involving animals were approved by and performed according to guidelines of the Institutional Animal Care and Use Committee of Mayo Foundation. For the safety experiment, female athymic mice were given 2 x 10^6 ^SKOV3ip.1 FLuc or a cocktail of SKOV3ip.1 and MSC (2 × 10^6 ^of each cell type) intraperitoneally. For imaging, mice were given i.p. injections of 150 mg/kg D-luciferin 10 minutes before imaging for FLuc activity. Whole abdominal bioluminescence signals reflecting tumor burden (FLuc) were quantitated using the Living Image 2.60 software according to manufacturer's protocol. Mice were euthanized when they developed ascites, lost more than 20% of body weight or if they developed a large injection site subcutaneous tumor of more than 10% body weight. All mice were euthanized at day 90 and Kaplan-Meier survival curves were plotted. The log-rank test was used to examine the significance of differences in the survival between groups. GraphPad Prism and JMP v9.0 were used for the statistical calculations. *P* < 0.05 was considered significant.

For the tumorigenicity study, 30 SCID beige mice were divided into three cohorts of ten. The test articles were three separate lots of high passage hMSC collected and purified from adipose tissue of three healthy donors. Mice receiving hMSC were injected subcutaneously on the right hind flank with approximately 2×10^7 ^hMSC/200μL saline and those injected i.p. received approximately 4×10^7 ^hMSC/400μL saline. One mouse was used as a control and received 5×10^6 ^SKOV3 tumor cells in 100 μL saline subcutaneously. Three mice were administered hMSC subcutaneously and the remaining six were administered hMSC i.p. Mice were monitored and tumors, if present, were measured and recorded. Body weights were collected. All mice were euthanized at or around day 90 and necropsied. Another cohort of athymic mice received 10^6 ^SKOV3ip.1 (n = 15 mice) or a cocktail of 10^6 ^SKOV3ip.1 and 10^6 ^MSC (n = 13 mice) ip. Survival curves were plotted and compared.

For the therapy experiment, female athymic mice (5–6 weeks of age; Harlan Laboratories; Indianapolis, IN) were injected i.p. with 2×10^6 ^SKOV3ip.1 cells stably expressing Firefly Luciferase (SKOV3ip.1-FLuc). Mice were imaged at day 7 using the Xenogen IVIS 200 bioluminescent imaging system (Xenogen, Alameda, CA). One day before treatment, MSC were pre-infected with MV-NIS (MOI 4.0) for two hours, virus was removed and the cells were frozen in aliquots in liquid nitrogen and stored until use. On day 0 a portion of MSC were irradiated at 20 Gray before infection. MSC, irradiated and non-irradiated, were infected with MV-NIS (MOI 4.0) for 2 h after which virus was removed. All MV-MSC were combined with neutralizing serum for 30 minutes at 37°C. On day 0, all mice were passively immunized with an i.p. injection of measles immune human pooled AB serum (Valley Biomedical, Winchester, VA) 2 h before therapy. The anti-MV titer of the purchased human AB serum was 300 EU/ml as determined by the Mayo Clinic Serology Clinical Laboratory using their routine measles antibody assay (Diamedix). In humans, a titer of > 20 EU/ml is considered to be measles immune. The full plaque reduction neutralization titer of the serum is 1:256 against 250 TCID_50 _of MV-GFP on Vero cells [33]. Mice were injected with the 3 different infected MSC platforms, naked virus (MV-NIS, 10^5 ^viral particles per dose), or saline i.p. For imaging, mice were given i.p. injections of 150 mg/kg D-luciferin (Xenogen) 10 minutes before imaging for firefly luciferase (FLuc) activity. Whole abdominal bioluminescence signals reflecting tumor burden (FLuc) were quantitated using the Living Image 2.60 software (Xenogen) according to manufacturer's protocol. For SPECT-CT imaging, mice received cells i.p., anesthesized by 2% isoflurane inhalation and imaged on a small animal micro-SPECT-CT machine (Gamma Medica, Northridge, CA). Mice were euthanized when they developed ascites, lost more than 20% of body weight or if they developed a large injection site subcutaneous tumor of more than 10% body weight. All mice were euthanized at day 90 and Kaplan-Meier survival curves were plotted. The log-rank test was used to examine the significance of differences in the survival between groups. GraphPad Prism (GraphPad Software, San Diego, CA) was used for the statistical calculations. *P* < 0.05 was considered significant.

## Competing interests

SJR and KWP are cofounders of Imanis Life Sciences (Rochester, MN). ABD is a shareholder of Mill Creek Life Sciences, (Rochester, MN).

## Authors’ contribution

EKM carried out the experimental studies and drafted the manuscript. GB isolated and characterized the MSC. SCD and AM performed the surgeries, harvested adipose tissues and ovarian tissues. KLK participated in the X-Celligence migration assays. MJF, SJR, EG and AB participated in study design. KWP conceived the study, performed analysis and drafted the manuscript. All authors read and approved the final manuscript.
